# Integrative Multi-Omics Profiling of Dynamic Body Mass Index–Systolic Blood Pressure Trajectories in Obesity for Precision Risk Stratification of Heart Failure Subtypes

**DOI:** 10.3390/biomedicines14071473

**Published:** 2026-06-29

**Authors:** Ya-Jie Zhai, Qun-Wei Ma, Xing-Jian Zhang, Xue-Qing He, Guang Wang, Jia Liu

**Affiliations:** Department of Endocrinology, Beijing Chao-Yang Hospital, Capital Medical University, Beijing 100020, China

**Keywords:** group-based multi-trajectory modeling, risk stratification, prognosis, heart failure, plasma proteomics, polygenetic risk score

## Abstract

**Background:** Obesity is an important risk factor for heart failure (HF). The dynamic heterogeneity of HF risk and its interaction with blood pressure changes among individuals with obesity remain unclear. This study aimed to improve risk assessment and support precision medicine by evaluating joint longitudinal trajectories of body mass index (BMI) and systolic blood pressure (SBP). **Methods:** We included 14,469 participants with BMI ≥ 30 from the UK Biobank. Group-based multi-trajectory modeling identified joint BMI-SBP trajectories. After inverse probability of treatment weighting, associations between trajectory groups and overall HF and its subtypes were evaluated, and potential mechanisms were explored using polygenic risk score (PRS) and plasma proteomic characteristics. **Results:** Four distinct trajectory patterns were identified: mild obesity with mild hypertension trajectory (MOMH), severe obesity with high-normal blood pressure progression trajectory (SOHN; BMI approximately 37.0 kg/m^2^, SBP increasing from approximately 131 to 138 mmHg), moderate obesity with high-normal blood pressure progression trajectory (MOHN; BMI approximately 33.0 kg/m^2^, SBP increasing from approximately 132 to 139 mmHg), and mild obesity with moderate-to-severe hypertension improvement trajectory (MOMSH). The SOHN group exhibited the highest risk of overall HF (HR=3.76). MOMSH and MOMH were associated with higher HFpEF risk (HRs: 2.68–2.70), whereas MOHN showed the lowest HF risk (HR = 1.82). These trajectory-based subtypes displayed heterogeneity in genetic susceptibility and plasma proteomic characteristics. **Conclusions:** Dynamic trajectory-based phenotyping identifies distinct obesity-BP patterns associated with differential HF risk and distinct nutritional and metabolic profiles, offering a more informative framework than static indicators for risk assessment.

## 1. Introduction

Heart failure (HF) is a clinical syndrome arising from a variety of cardiac diseases that cause structural and functional abnormalities, leading to impaired ventricular filling or ejection [1]. Clinically, HF can present with reduced left ventricular ejection fraction (LVEF), including HF with reduced ejection fraction (HFrEF) and HF with mildly reduced ejection fraction (HFmrEF), or with preserved ejection fraction (HFpEF) [2]. Globally, an estimated 64 million individuals are affected by HF, with a lifetime risk of approximately 24% [3]. The high morbidity and mortality associated with HF impose substantial economic burdens on both society and affected families [4]. Obesity is a major risk factor for the development and progression of HF and is frequently accompanied by elevated blood pressure [5]. However, the phenotypic heterogeneity of HF risk among individuals with obesity and its interactive mechanisms with dynamic blood pressure changes remain inadequately elucidated.

There is significant individual heterogeneity in obese people, which leads to significant differences in the dynamic evolution of HF and the susceptibility to cardiovascular complications [6]. Conventional diagnostic criteria based on static thresholds have limited capacity to dynamically predict HF risk and fail to integrate the synergistic, dynamic changes of obesity and hypertension. Therefore, constructing a refined risk stratification model grounded in the joint dynamic trajectories of obesity and blood pressure holds significant potential for identifying high-risk HF subgroups, enabling early individualized interventions, and reducing obesity-associated HF burden.

Compared with traditional risk stratification models based on baseline indicators, Group-based multi-trajectory modeling (GBMTM) offers notable advantages in capturing the temporal dynamics of disease progression. Unsupervised longitudinal trajectory analyses have already identified prognostic subgroups with distinct dynamic phenotypes in conditions such as atherosclerosis, depression, and chronic pulmonary diseases [7,8,9]. To systematically elucidate the dynamic heterogeneity of HF risk in the obese population and establish an early dynamic trajectory-based risk stratification framework, this study employs GBMTM to identify joint growth trajectories of body mass index (BMI) and systolic blood pressure (SBP), and to characterize HF high-risk subgroups associated with different trajectories.

## 2. Materials and Methods

### 2.1. Study Design and Population

This study utilized data from the UK Biobank (UKB; application number 425612), a prospective cohort study that recruited over 500,000 community-dwelling participants aged 40–69 years from 22 assessment centers across England (89%), Scotland (7%), and Wales (4%). Baseline assessments were conducted between 2006 and 2010. UKB has obtained ethical approvals from the North-West Multi-Centre Research Ethics Committee, the Participant Information Advisory Group, and the Community Health Index Advisory Group. We included obese participants with complete data and BMI ≥ 30 kg/m^2^. Exclusion criteria were: presence of HF at baseline (including HFmrEF, HFpEF, or HFrEF), fewer than two measurements of BMI or SBP during follow-up, missing baseline BMI or SBP data, or a history of cancer at baseline. After screening, 14,469 UKB participants met the requirements for trajectory analysis. To identify factors associated with obesity- and blood pressure trajectory groups, a healthy control cohort with normal SBP and BMI was also included; ultimately, 28,601 participants were included in the longitudinal analysis. The selection flowchart is detailed in the Supplementary Material, Figure S1.

### 2.2. Clinical Outcomes

This study evaluated HF clinical outcomes across participants in different trajectory groups. HF diagnosis was identified using ICD-10 code I50 within the UK Biobank data, encompassing primary care records, death registry data, and self-reported HF diagnoses. To further specify HF subtypes, we integrated LVEF data derived from cardiovascular magnetic resonance (CMR) imaging. LVEF was extracted from raw CMR images using automated analysis software. Participants had complete CMR parameters, including LVEF, left ventricular end-diastolic volume (LVEDV), LV stroke volume, cardiac output, and left ventricular global longitudinal strain; abnormal values with LVEDV ≥ 500 mL were excluded. Based on LVEF [2], we further examined HF clinical outcomes across subtypes: (1) HF with preserved EF (HFpEF; LVEF ≥ 50%), (2) HF with mildly reduced EF (HFmrEF; LVEF 41–49%), and (3) HF with reduced EF (HFrEF; LVEF ≤ 40%). Participants included HF cases diagnosed before imaging and HF cases diagnosed after imaging assessment. This mixed approach enabled comprehensive capture of HF participants, given that the assessment age of UK Biobank participants is typically lower than the typical age of HF onset. Follow-up time was defined as the interval from the baseline recruitment date to the date of death or the date of last follow-up.

### 2.3. Data Collection and Measurements

This study systematically collected a variety of covariates. Socio-demographic factors included sex (male, female), age, race (white, non-white), and educational attainment (high school or below, college or above). Lifestyle factors encompassed smoking status (current smoker, former smoker, never smoker) and alcohol use (current drinker, former drinker, never drinker). Anthropometric measurements included systolic and diastolic blood pressure. Laboratory biomarkers included C-reactive protein (CRP), creatinine, glycated hemoglobin (HbA1c), estimated glomerular filtration rate (eGFR), high-density lipoprotein cholesterol (HDL-C), low-density lipoprotein cholesterol (LDL-C), and triglycerides (TG). The eGFR was calculated using the CKD-EPI creatinine equation [10]. The specific field IDs for all variables are provided in Supplementary Material Table S1.

### 2.4. Latent Trajectory Analysis

To identify distinct joint developmental trajectories of BMI and SBP, we employed GBMTM. This approach integrates latent class analysis with multilevel modeling, enabling simultaneous characterization of longitudinal patterns across multiple correlated variables and identification of latent subgroups sharing similar trajectory features [11]. GBMTM fits finite mixture models using maximum likelihood estimation to identify trajectory subgroups characterized by distinct polynomial functions of time, providing asymptotically unbiased parameter estimates under the assumption that missing data are missing at random [12]. In this study, BMI and SBP were modeled as joint outcome variables, with follow-up time serving as the time scale. A two-stage procedure was implemented to determine the optimal trajectory model. First, starting from a single-trajectory model, we sequentially fitted models specifying one to four trajectory groups and compared model fit using the Bayesian Information Criterion (BIC), with lower BIC values indicating superior fit [13]. After establishing the optimal number of trajectory groups, we further refined the polynomial order for each trajectory. The final model was chosen by balancing goodness-of-fit and parsimony, with quality assessed by: (1) average posterior probability (AvePP) ≥ 0.7; (2) odds of correct classification (OCC) ≥ 5; and (3) sufficient sample size within each trajectory group [13]. The optimal number of subphenotypes was four. Trajectory modeling included participants with complete data for at least two follow-up time points. Model estimation was performed using the gbmt package in R.

### 2.5. Inverse Probability of Treatment Weighting

Given the substantial imbalance in sample sizes between trajectory groups and the healthy control group, we employed inverse probability of treatment weighting (IPTW) to fully utilize the available data and mitigate potential confounding bias. IPTW weights were calculated based on a comprehensive set of covariates, including age, sex, ethnicity, educational level, and factors showing significant intergroup differences at baseline, such as smoking status, alcohol consumption, HbA1c, LDL, creatinine, eGFR, and CRP. Propensity scores were estimated using generalized boosted models, and IPTW was implemented using the Weight It package (version 1.0.0). In order to stabilize the weight distribution, the extreme weights (>10) were truncated by 1% and 99% quantiles. Covariate balance before and after weighting was assessed using standardized mean differences. Improvements in covariate balance were visualized using Love plots.

### 2.6. Polygenic Risk Scores Calculation

Polygenic risk scores (PRS) quantify an individual’s genetic susceptibility to specific diseases or phenotypes by aggregating the cumulative effects of multiple genetic variants. In this study, PRS for BMI, hypertension, cardiovascular disease (CVD), coronary artery disease (CAD), atrial fibrillation, type 2 diabetes mellitus (T2DM), and stroke were obtained from the UK Biobank PRS Release dataset. Additionally, this study calculated the enhanced PRS [14]. In the construction process, not only the genetic data of external independent samples were integrated, but also the training samples of another independent subgroup in the British Biobank were included, which significantly improved the prediction accuracy of the model and the generalization ability across populations. By leveraging multi-source data, enhanced PRS more effectively capture genetic variant signals associated with specific phenotypes, thereby providing a more reliable measure of genetic susceptibility for subsequent analyses. All PRS values were normalized using z-score standardization. Differences in PRS distributions across trajectory groups were assessed using the Kruskal–Wallis test, and Bonferroni method was used to perform multiple test correction on the results of pairwise comparison between groups.

### 2.7. Proteomics Processing

We utilized plasma proteomics data from the UK Biobank Pharma Proteomics Project (UKB-PPP) measured on the Olink Explore 3072 platform. The dataset spans approximately 54,000 participants. Data processing included sample selection, data import, exclusion of individuals who withdrew consent or had data anomalies, removal of samples failing quality control, and detection of potential sample swaps [15,16]. Following rigorous quality control and normalization, we obtained each participant’s Normalized Protein Expression (NPX), a log2-transformed relative protein abundance metric defined by Olink. The final sample size for proteomic analysis is provided in Table S5. Differential expression analysis was conducted in R using the limma package. After transposing the NPX matrix, linear models were constructed, and variance shrinkage estimation was performed using the empirical Bayes (eBayes) algorithm. Proteins with Benjamini–Hochberg adjusted *p*-values (adj.*p*.Val) < 0.05 and |log_2_ fold change| > 0.3 were considered significantly differentially expressed. For identified proteins, Gene Ontology (GO) and Kyoto Encyclopedia of Genes and Genomes (KEGG) pathway enrichment analyses were performed using the clusterProfiler package v4.10.1, with significance at *p* < 0.05. GO categories included Biological Process (BP), Molecular Function (MF), and Cellular Component (CC), with Benjamini–Hochberg correction for multiple testing. Results were visualized using ggplot2 v4.0.3. Volcano plots were generated to display the overall distribution of differentially expressed proteins, with the top 10 most significantly up- and down-regulated proteins annotated. Enrichment results were presented as bubble plots, where dot size represents the number of enriched genes and color intensity reflects log_10_ (adjusted *p*-value), which visually displayed the specific biological functions and pathway characteristics of each trajectory group.

### 2.8. Statistical Analysis

To assess the stability of trajectory-derived subgroups, participants were classified into four subgroups as described above, and group differences in clinical characteristics were compared. Categorical variables were reported as counts and percentages and compared using the chi-squared (χ^2^) test. Continuous variables were presented as mean ± standard deviation; between-group comparisons were conducted using one-way analysis of variance (ANOVA) or the Kruskal–Wallis test, as appropriate to data distribution. We applied IPTW-adjusted Cox proportional hazards models to estimate the risk of incident overall HF, HFpEF, HFmrEF, and HFrEF across trajectory groups, using the healthy control group as the reference. IPTW weights were incorporated to control for confounding, and cumulative incidence curves were plotted. All analyses were conducted in R (version 4.4.0). All tests were two-sided, with a significance level of *p* < 0.05.

## 3. Results

### 3.1. GBMTM Identified Four Distinct Trajectory Groups

**Figure 1:**
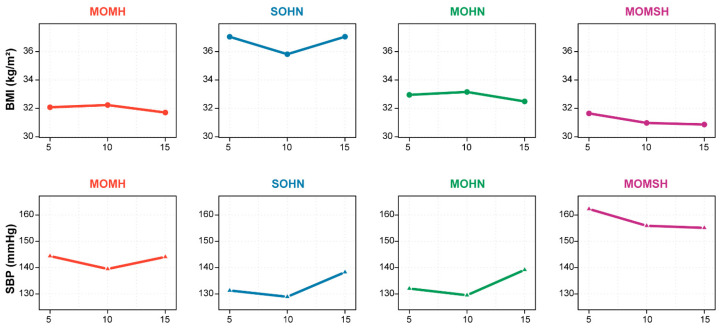
Different phenotypes based on the combined dynamic trajectory of BMI and systolic blood pressure. MOMH, mild obesity with mild hypertension trajectory; SOHN, severe obesity with high-normal blood pressure progression trajectory; MOHN, moderate obesity with high-normal blood pressure progression trajectory; MOMSH, mild obesity with moderate-to-severe hypertension improvement trajectory.

GBMTM identified four distinct joint developmental trajectories of BMI and SBP (Figure 1). Model fit indices demonstrated robust classification performance (see Supplementary Table S2), with an AvePP of 0.92 and an OCC of 37.37. Additionally, each trajectory group comprised more than 20% of the study population (see Supplementary Figure S2), indicating high stability of the identified trajectory structure. A total of 14,469 participants were included in the trajectory analysis, and baseline clinical characteristics of each dynamic trajectory group are presented in Table 1. The mild obesity with mild hypertension trajectory (MOMH) comprised 3596 participants (24.9%) and was characterized by older age, higher proportion of males, highest prevalence of current alcohol consumption, and higher triglyceride levels. The severe obesity with high-normal blood pressure progression trajectory (SOHN) included 3660 participants (25.3%), predominantly female, with the highest levels of HbA1c and CRP. The moderate obesity with high-normal blood pressure progression trajectory (MOHN) consisted of 3755 participants (26.0%), exhibiting intermediate levels of HbA1c and CRP. The mild obesity with moderate-to-severe hypertension improvement trajectory (MOMSH) comprised 3458 participants (23.9%), characterized by the oldest age, highest proportion of males, highest prevalence of former smokers, highest lipid and creatinine levels, and lowest eGFR.

### 3.2. Inverse Probability of Treatment Weighting Analysis

In order to evaluate the independent association between different BMI-SBP combined trajectory groups and the risk of HF, this study used IPTW method to balance the baseline covariates of different trajectory groups and healthy control group. Following IPTW application, covariate balance across groups was substantially improved. As illustrated in Figure S3a, the propensity score distribution between the weighted adjusted trajectory population and the healthy control group tended to be consistent. Figure S3b demonstrates that after IPTW adjustment, standardized mean differences (SMD) for all covariates were reduced to below 15%. Further analyses (see Supplementary Tables S3 and S4) revealed that following IPTW adjustment, there was no statistical difference in the comparison of most covariates between groups (all *p* > 0.05), which effectively eliminated the confounding bias.

### 3.3. Risk of HF Subtypes

**Figure 2:**
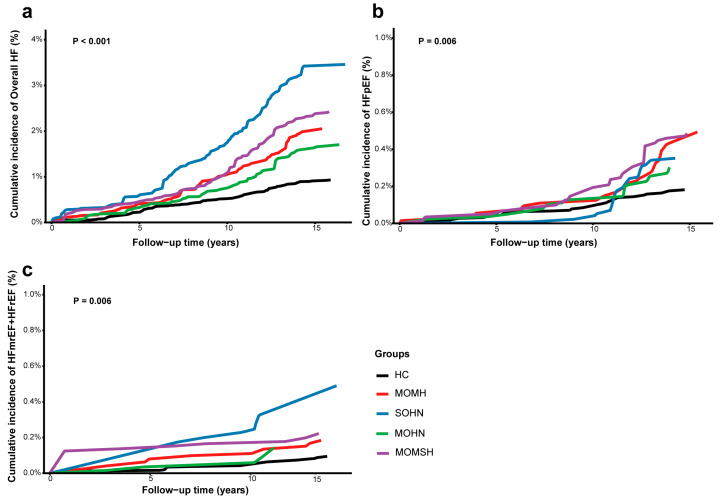
The incidences of complications in different groups. (**a**) Cumulative incidence of Overall HF; (**b**) Cumulative incidence of HFpEF; (**c**) Cumulative incidence of HFmrEF + HFrEF; MOMH, mild obesity with mild hypertension trajectory; SOHN, severe obesity with high-normal blood pressure progression trajectory; MOHN, moderate obesity with high-normal blood pressure progression trajectory; MOMSH, mild obesity with moderate-to-severe hypertension improvement trajectory; HF, heart failure; HFpEF, heart failure with preserved ejection fraction; HFmrEF, heart failure with mid-range ejection fraction; HFrEF, heart failure with reduced ejection fraction.

The risk of incident HF across subgroups was evaluated using IPTW-adjusted Kaplan–Meier analysis. As shown in Figure 2 and Table 2, the SOHN exhibited the highest cumulative incidence of overall HF and HFmrEF combined with HFrEF, with weighted hazard ratios significantly increased by 3.76–5.20-fold (all *p* < 0.05). Notably, the MOMSH group demonstrated the second-highest cumulative incidence of overall HF (HR = 2.63, 95% CI: 1.99–3.47, *p* < 0.001). Among patients with HFpEF, both the MOMSH group and the MOMH group showed similar high-risk levels (all *p* < 0.05). In contrast, the MOHN showed the lowest cumulative incidence of overall HF, and its weighted risk increased significantly by 1.82 times (*p* = 0.003).

### 3.4. Polygenic Risk Scores

Table 3 and Figure S4 compare disease-specific PRS across distinct dynamic trajectory subgroups to explore the distributional characteristics of genetic susceptibility. Results revealed significant intergroup differences in genetic risk for multiple diseases (*p* < 0.05). The MOMSH group exhibited the highest PRS for hypertension (0.25 ± 0.90), T2DM (0.05 ± 0.99), AF (0.16 ± 1.01), CVD (0.07 ± 1.00), and stroke (0.25 ± 0.92), indicating substantially enhanced genetic susceptibility. The SOHN group demonstrated the most prominent BMI-related genetic risk (0.70 ± 0.99), significantly exceeding that of other subgroups. The MOMH and MOHN groups displayed intermediate PRS levels across most diseases, with the SOHN group showing relatively elevated genetic risk for atrial fibrillation (0.11 ± 0.99). In contrast, the healthy control group consistently exhibited lower genetic risk across multiple diseases, suggesting an overall genetic protective effect.

### 3.5. Proteomic Analysis

**Figure 3:**
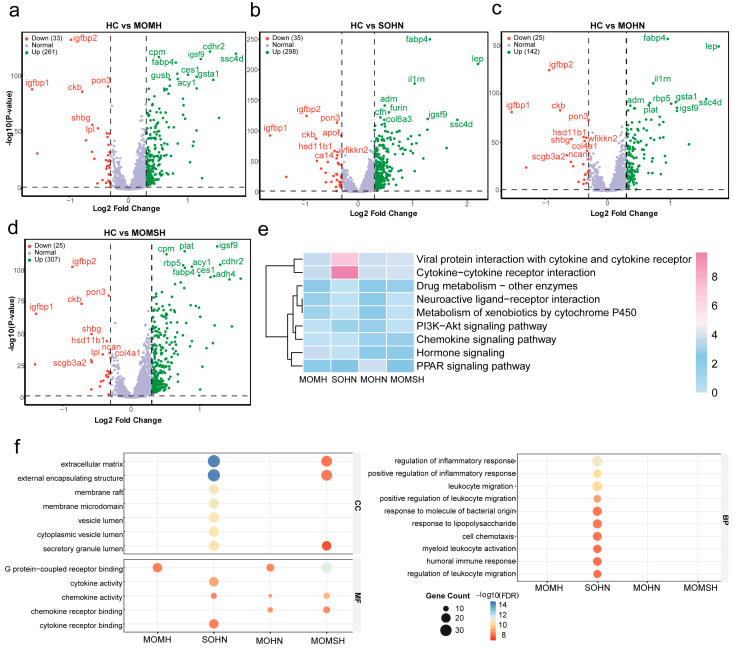
Volcano maps and enrichment analysis of differential plasma proteomics. (**a**–**d**) Volcano maps; (**e**) KEGG analysis; (**f**) GO analysis. GO, gene ontology; KEGG, Kyoto encyclopedia of genes and genomes; MF, Molecular Function; CC: Cellular Component; BP, biological Process; HC, health control; MOMH, mild obesity with mild hypertension trajectory; SOHN, severe obesity with high-normal blood pressure progression trajectory; MOHN, moderate obesity with high-normal blood pressure progression trajectory; MOMSH, mild obesity with moderate-to-severe hypertension improvement trajectory.

Figure 3 shows volcano plots depicting differentially expressed proteins between each trajectory subgroup and the healthy control group. Through comparative plasma proteomic profiling coupled with GO and KEGG pathway enrichment analyses, we identified distinct molecular signatures characterizing each subgroup. In the MOMH group, insulin-like growth factor-binding proteins 2 (IGFBP2) and 1 (IGFBP1) were significantly downregulated, whereas carboxypeptidase M (CPM) and fatty acid-binding protein 4 (FABP4) were substantially upregulated. Enriched proteins were primarily involved in amino acid metabolism, with significant enrichment in the PPAR signaling pathway and cytokine-cytokine receptor interaction. The SOHN group exhibited marked upregulation of leptin (LEP) and interleukin-1 receptor antagonist (IL1RN), along with increased adrenomedullin (ADM) expression. The related functions converged on inflammatory response regulation and leukocyte migration, with significant enrichment in the TNF signaling pathway. In the MOHN group, retinol-binding protein 5 (RBP5) was prominently upregulated. Associated pathways involved cellular chemotaxis and positive regulation of JAK-STAT signaling, with predominant enrichment in cytochrome P450-mediated xenobiotic metabolism. The MOMSH group demonstrated significant upregulation of tissue-type plasminogen activator (PLAT) and protocadherin-24 (CDHR2), accompanied by increased alcohol dehydrogenase 4 (ADH4) expression. Functional characterization revealed enrichment in antimicrobial humoral immune responses, leukocyte migration, and receptor-mediated endocytosis, implicating chemokine signaling pathways and hormone-mediated signaling processes.

## 4. Discussion

The prevalence and burden of HF are increasing in the context of the increasing number of obese people worldwide [17]. Conventional HF risk assessment tools, such as the 10-TAGA instrument and the GWTG-HF score [18,19], primarily rely on static risk factors, and have limited ability to identify complex dynamic trajectories and phenotypic heterogeneity of HF in obese people. In this study, we innovatively applied GBMTM to analyze joint longitudinal trajectories of BMI and SBP among individuals with obesity, identifying for the first time four distinct cardiometabolic dynamic phenotypes. Each trajectory subgroup was characterized by unique temporal progression patterns, genetic backgrounds, plasma protein profiles, and distinct associations with HF risk profiles. These findings establish a critical foundation for precision prevention of HF based on cardiometabolic dynamic phenotyping.

Previous studies have demonstrated that the synergistic effect of BMI and blood pressure is more predictive of cardiocerebrovascular outcomes than a single indicator [20,21]. Furthermore, HF exhibits significant temporal heterogeneity across different cardiometabolic exposure patterns [22,23,24], suggesting that static metabolic indicators are insufficient to capture the dynamic evolutionary characteristics of HF. Meanwhile, conventional risk models have inherent limitations in quantifying heterogeneous patterns of HF development. For the first time, this study applied a group-based multiple trajectory model in obese people to verify and expand the above findings. We identified four distinct joint BMI-SBP dynamic trajectories, among which the SOHN and the MOMSH exhibited markedly abnormal glucolipid metabolic and inflammatory states, accompanied by a high risk of specific HF subtypes. Therefore, integrating dynamic obesity-hypertension trajectories into HF risk stratification frameworks enables precision stratification that transcends the limitations of static BMI and SBP thresholds, facilitating early identification of high-risk individuals and targeted interventions.

IPTW-adjusted Kaplan–Meier analysis revealed significant differences in HF risk across different combined trajectories. The SOHN group exhibited the highest cumulative incidence of overall HF, whereas the MOHN group, despite demonstrating similar dynamic blood pressure patterns, showed the lowest overall HF risk among all trajectories, suggesting that obesity is an independent risk factor for HF under the same blood pressure exposure pattern, and there is a direct relationship between obesity and cardiac remodeling and cardiac dysfunction [25]. Previous studies have reported associations between obesity, visceral fat accumulation, and the occurrence and progression of HFpEF [25,26]. This is consistent with our results: MOMSH group and MOMH group showed the highest and similar risk levels in HFpEF, indicating that improvements in blood pressure trajectories do not completely offset obesity-related HF risk. Notably, the blood pressure improvement in the MOMSH group may partly reflect antihypertensive treatment effects, and the persistent HFpEF risk in this group suggests that pharmacological blood pressure lowering alone may be insufficient to fully reverse HF risk. Among the three groups with similar obesity levels but progressively increasing blood pressure exposure patterns (MOMH, MOHN, and MOMSH), overall HF risk increased incrementally with elevated blood pressure exposure. This underscores effective blood pressure control as a critical target for HF prevention in obese populations. However, despite improved blood pressure trajectories in the MOMSH group, HFpEF risk remained substantially elevated, suggesting that blood pressure-lowering interventions alone are insufficient to fully reverse HF risk. Therefore, combining weight reduction with blood pressure management may represent a more effective strategy for mitigating HF risk.

Genetic susceptibility analysis further validated the trajectory-based stratification at the biological level. The MOMSH group exhibited the highest PRS for hypertension, stroke, and CVD, which closely aligned with its history of hypertension exposure and high-risk HFpEF phenotype, suggesting that genetic predisposition to vascular-related traits may be associated with the observed HFpEF risk pattern [27]. The SOHN group demonstrated the most prominent BMI-related genetic risk, which was associated with elevated HF risk, consistent with previous reports linking genetic obesity predisposition to HF-related pathways such as volume overload and neurohormonal activation [28,29]. This correspondence among genetic risk, trajectory patterns, and HF subtypes provides a basis for considering dynamic trajectory-based stratification in conjunction with genetic background information. For individuals exhibiting both the MOMSH phenotype and high-risk PRS profiles, early implementation of weight management and blood pressure interventions is warranted to interrupt the progression of HF driven by gene-environment interactions.

Proteomic analysis revealed distinct molecular signatures characterizing each BMI-SBP joint trajectory subgroup. In the MOMSH group, 307 proteins were significantly upregulated, of which PLAT, a key activator of the fibrinolytic system, was upregulated; this finding is consistent with previous reports linking PLAT to fibrinolytic processes potentially related to endothelial injury responses [30], while CDHR2 upregulation was associated with cell–cell adhesion and inflammatory cell recruitment processes [31]. These findings may suggest that vascular endothelial damage and chronic inflammation markers are associated with the elevated HFpEF risk observed in this subgroup, although causal relationships cannot be inferred from this observational study. The SOHN group exhibited marked upregulation of LEP, IL1RN, and ADM, with functional enrichment in inflammatory response regulation and leukocyte migration, predominantly involving the TNF signaling pathway. As an adipose tissue-derived hormone, elevated LEP is consistent with previous observations linking increased leptin levels to visceral adiposity expansion and metabolically associated inflammatory states [32]; IL1RN upregulation is consistent with patterns observed in IL-1β-mediated inflammatory processes [33]; and increased ADM is consistent with patterns of neurohormonal activation reported in volume overload states [34]. Collectively, these proteins are enriched in pathways related to cardiometabolic inflammation, which may partly explain the elevated HF risk observed in this subgroup. However, these proteomic associations are exploratory and require validation in an independent cohort. In contrast, the MOHN group demonstrated significant upregulation of RBP5, with associated pathways involving cellular chemotaxis and positive regulation of JAK-STAT signaling. RBP5 elevation has been linked to retinol metabolism and antioxidant defense pathways [35], which may reflect differences in metabolic profiles compared with other trajectory groups, consistent with the relatively lower HF risk observed in this subgroup. The specific protein expression patterns of each trajectory subgroup are consistent with their dynamic phenotype–HF subtype associations, supporting the potential utility of plasma protein biomarkers as complementary tools in dynamic risk stratification frameworks. In addition, our results likely reflect cumulative cardiometabolic injury from long-term exposure to elevated BMI and SBP. The increased HF risk in the MOMSH and SOHN groups may be related to persistent vascular remodeling, arterial stiffness, and obesity-related metabolic stress [36,37,38]. By contrast, the lower risk in the MOHN group may indicate a more favorable metabolic state, although this should not be viewed as benign obesity. These findings are consistent with the obesity paradox and HFpEF phenotyping literature [39,40], and they further support the value of trajectory-based approaches for capturing risk beyond static measurements.

Based on the distinct BMI-SBP joint trajectory subgroups identified, this study generates hypotheses for potential stratification approaches. The MOHN group, exhibiting the lowest overall HF risk and a protein profile suggestive of metabolic adaptation patterns, may represent a subgroup in which lifestyle maintenance to prevent progression to severe obesity, coupled with regular monitoring of cardiac function, could be considered as a preventive approach. The MOMSH group demonstrated elevated HFpEF risk, characterized by the oldest age, poorest renal function, highest lipid levels, and most pronounced genetic susceptibility to hypertension, with proteomic profiling revealing PLAT and CDHR2 upregulation and enrichment of chemokine signaling pathways. For the MOMSH group, hypothetical management strategies might strengthen vascular protection and anti-fibrosis intervention on the basis of strict blood pressure control. These hypotheses should be tested in prospective interventional studies, and routine monitoring of diastolic function and renal function may be warranted in this subgroup. Given the observational nature of our proteomic findings, the optimal management strategy for this population remains to be determined and requires prospective validation. The SOHN group exhibited the highest overall HF risk, with elevated LEP, IL1RN, and ADM suggesting metabolically driven inflammation and volume overload activation. These findings raise the hypothesis that interventions targeting weight management and combined with anti-inflammatory approaches, might modify HF risk in this subgroup; however, this hypothesis also requires confirmation through prospective interventional studies. These trajectory-based individualized strategies provide an evidence-based foundation for precision prevention of HF in obese populations, with the potential to reduce HF incidence and improve clinical outcomes.

## 5. Limitations

There are some limitations in this study. First, as an observational cohort study, despite employing methods such as IPTW to control for confounding, the interference of residual confounding and drug treatment cannot be completely excluded. UKB image participants limit the spread of the disease. Second, a although this study found genetic susceptibility and proteomics characteristics, the potential causal mechanisms underlying these associations require further verification through animal models. Third, the study population was primarily derived from the UK Biobank cohort, which has a recognized selection bias. Fourth, since proteomics comparisons are only for healthy controls, the interpretation of specific molecular features that may distinguish HFpEF-and HFrEF-tendency trajectories is limited. Therefore, the generalization of the research results needs to be verified in different populations. Future research should integrate multicenter cohorts, sex-stratified designs, longitudinal multi-omics profiling, medication records, and functional validation studies to verify and optimize the dynamic trajectory classification model, thereby advancing precision prediction and individualized intervention strategies.

## 6. Conclusions

Through integrative multi-omics (GBMTM, PRS, and plasma proteomics), we identified four BMI-SBP trajectory patterns associated with differing risks of HF and distinct genetic and proteomic signatures. The SOHN trajectory showed highest obesity genetic risk with inflammatory activation (LEP, IL1RN, ADM), associated with HFrEF/HFmrEF, while MOMSH displayed hypertension genetic susceptibility and endothelial injury signatures (PLAT, CDHR2), conferring elevated HFpEF risk. These dynamic phenotypes enable precision prevention strategies tailored to specific molecular mechanisms, advancing HF risk assessment from static thresholds to integrative, trajectory-based stratification frameworks.

## Figures and Tables

**Table 1:** Baseline characteristics of different groups.

Characteristics	MOMH	SOHN	MOHN	MOMSH	*p*
n	3596	3660	3755	3458	
Age, years	55.12 ± 7.28	52.79 ± 7.32	53.40 ± 7.27	57.52 ± 6.82	<0.001
Sex, n (%)					<0.001
Female	1471 (40.9)	2332 (63.7)	1968 (52.4)	1344 (38.9)	
Male	2125 (59.1)	1328 (36.3)	1787 (47.6)	2114 (61.1)	
Ethnicity, n (%)					<0.001
White	3446 (95.8)	3491 (95.4)	3589 (95.6)	3364 (97.3)	
Non-White	150 (4.2)	169 (4.6)	166 (4.4)	94 (2.7)	
Drinking status, n (%)					<0.001
Never	85 (2.4)	149 (4.1)	123 (3.3)	98 (2.8)	
Previous	90 (2.5)	148 (4.0)	99 (2.6)	75 (2.2)	
Current	3419 (95.1)	3359 (91.9)	3532 (94.1)	3282 (95.0)	
Smoking status, n (%)					<0.001
Never	1970 (55.0)	2060 (56.5)	2106 (56.2)	1846 (53.6)	
Previous	1384 (38.6)	1316 (36.1)	1364 (36.4)	1428 (41.5)	
Current	231 (6.4)	269 (7.4)	279 (7.4)	170 (4.9)	
Education, n (%)					0.336
College	415 (11.5)	386 (10.5)	421 (11.2)	425 (12.3)	
Other levels	3164 (88.0)	3262 (89.1)	3319 (88.4)	3017 (87.2)	
Unknown	17 (0.5)	12 (0.3)	15 (0.4)	16 (0.5)	
HbA1c, mmol/mol	36.48 ± 6.44	37.67 ± 8.15	36.70 ± 7.28	36.93 ± 6.55	<0.001
LDL, mmol/L	3.66 ± 0.86	3.52 ± 0.84	3.61 ± 0.86	3.72 ± 0.90	<0.001
HDL, mmol/L.	1.29 ± 0.31	1.26 ± 0.29	1.28 ± 0.30	1.31 ± 0.32	<0.001
TG, mmol/L	2.16 ± 1.16	2.04 ± 1.08	2.12 ± 1.18	2.24 ± 1.22	<0.001
eGFR, ml/(min × 1.73 m^2^)	94.84 ± 12.35	95.65 ± 13.49	95.77 ± 12.66	93.64 ± 12.15	<0.001
CRP, mg/L	3.14 ± 4.11	4.63 ± 4.82	3.29 ± 4.19	3.02 ± 3.77	<0.001
Creatinine, umol/L	75.07 ± 14.26	71.48 ± 14.57	73.20 ± 14.31	75.32 ± 14.42	<0.001

Data are presented as mean ± standard deviation, or *n* (%). HbA1c, hemoglobin A1c; LDL, low-density lipoprotein; HDL, high-density lipoprotein; TG, triglycerides; CRP, C-reactive protein; eGFR, estimated glomerular filtration rate; MOMH, mild obesity with mild hypertension trajectory; SOHN, severe obesity with high-normal blood pressure progression trajectory; MOHN, moderate obesity with high-normal blood pressure progression trajectory; MOMSH, mild obesity with moderate-to-severe hypertension improvement trajectory. *p* <0.05.

**Table 2:** The risk of endpoint events in each heart failure subtype based on IPTW.

Groups	Overall HF		HFpEF		HFmrEF + HFrEF	
	HR (95% CI)	*p*	HR (95% CI)	*p*	HR (95% CI)	*p*
HC	Ref		Ref		Ref	
MOMH	2.22 (1.67–2.96)	<0.001	2.70 (1.47–4.98)	0.006	1.96 (0.77–5.01)	0.176
SOHN	3.76 (2.94–4.81)	<0.001	1.93 (0.97–3.83)	0.142	5.20 (2.55–10.62)	0.006
MOHN	1.82 (1.35–2.46)	0.003	1.67 (0.83–3.35)	0.204	1.51 (0.55–4.12)	0.492
MOMSH	2.63 (1.99–3.47)	<0.001	2.68 (1.43–5.02)	0.008	2.37 (0.96–5.87)	0.206

IPTW-adjusted hazard ratios (HR) and 95% confidence intervals as measures of relative effect. IPTW, inverse probability of treatment weighting; HC, health control; MOMH, mild obesity with mild hypertension trajectory; SOHN, severe obesity with high-normal blood pressure progression trajectory; MOHN, moderate obesity with high-normal blood pressure progression trajectory; MOMSH, mild obesity with moderate-to-severe hypertension improvement trajectory; HF, heart failure; HFpEF, heart failure with preserved ejection fraction; HFmrEF, heart failure with mid-range ejection fraction; HFrEF, heart failure with reduced ejection fraction.

**Table 3:** Polygenic risk score for different groups.

	MOMH	SOHN	MOHN	MOMSH	HC	*p*
Hypertension	0.05 ± 1.01	0.00 ± 1.03	−0.01 ± 1.00	0.25 ± 0.90	−0.27 ± 0.92	<0.001
BMI	0.29 ± 0.94	0.70 ± 0.99	0.40 ± 0.94	0.33 ± 0.95	−0.46 ± 0.99	<0.001
T2DM	−0.01 ± 0.99	0.04 ± 1.01	−0.01 ± 0.94	0.05 ± 0.99	−0.31 ± 0.99	<0.001
Stroke	0.02 ± 1.02	0.04 ± 1.01	0.02 ± 0.99	0.25 ± 0.92	−0.23 ± 0.95	<0.001
CVD	−0.07 ± 0.99	−0.12 ± 1.03	−0.04 ± 0.98	0.07 ± 1.00	−0.21 ± 1.02	<0.001
AF	0.04 ± 0.99	0.11 ± 0.99	0.06 ± 0.97	0.16 ± 1.01	0.03 ± 0.98	0.042

Polygenic risk scores are presented as mean ± standard. HC, health control; MOMH, mild obesity with mild hypertension trajectory; SOHN, severe obesity with high-normal blood pressure progression trajectory; MOHN, moderate obesity with high-normal blood pressure progression trajectory; MOMSH, mild obesity with moderate-to-severe hypertension improvement trajectory; BMI, body mass index; T2DM, type 2 diabetes mellitus; CAD, coronary artery disease; AF, atrial fibrillation; CAD, coronary artery disease.

## Data Availability

The data underlying this article are available in UK Biobank, at http://www.ukbiobank.ac.uk/, (accessed on 4 January 2026).

## Supplementary Materials

The following supporting information can be downloaded at: https://www.mdpi.com/article/10.3390/biomedicines14071473/s1, Figure S1. Flow chat of study. Figure S2. The pie chart of 4 trajectory subgroups. Figure S3. Evaluating covariate equilibrium pre- and post-IPTW adjustments. (a) Plot of standardized mean differences (SMD); (b) Density plot of propensity score distribution. Figure S4. Polygenic risk score for different groups. Table S1. The field IDs of all variables in UKB cohort. Table S2. Multivariate trajectory Group-Based Multi-Trajectory Modeling optimal model. Table S3. Baseline characteristics of participants before IPTW matching. Table S4. Comparison of covariates between groups after IPTW adjustment. Table S5. Sample size included in proteomic subgroup analysis.
