# Supplementary material for: Integrative Multi-Omics Profiling of Dynamic Body Mass Index–Systolic Blood Pressure Trajectories in Obesity for Precision Risk Stratification of Heart Failure Subtypes
Source: Biomedicines. 2026 Jun 29;14(7):1473. doi: 10.3390/biomedicines14071473 (PMC13404759; doi:10.3390/biomedicines14071473)
Supplement: Supplementary file 1 [file biomedicines-14-01473-s001.zip › biomedicines-4352554-supplementary.pdf]

Supplementary Material

Appendix to “Integrative Multi-Omics Profiling of Dynamic Body Mass Index–Systolic Blood Pressure Trajectories in Obesity for Precision Risk Stratification of Heart Failure Subtypes”

Contents

**Section S1: Flow chart of study participants ---
-3**

Figure S1. Flow chat of study participants ---3

**Section S2: Materials and methods ---
3**

Table S1. The field IDs of all variables in UKB cohort --3

**Section S3: Latent trajectory analysis --
-5**

Table S2. Multivariate trajectory Group-Based Multi-Trajectory Modeling optimal model ---5

Figure S2. The pie chart of 4 trajectory subgroups ---5

**Section S4: Results --
5**

Figure S3. Evaluating covariate equilibrium pre- and post-IPTW adjustments-------------------5

Table S3. Baseline characteristics of participants before IPTW matching -----------------------6

Table S4. Comparison of covariates between groups after IPTW adjustment --------------------7

Figure S4. Polygenic risk score for different groups--8

Table S5 Sample size included in proteomic subgroup analysis ----------------------------------8

Section S1: Flow chat of study participants

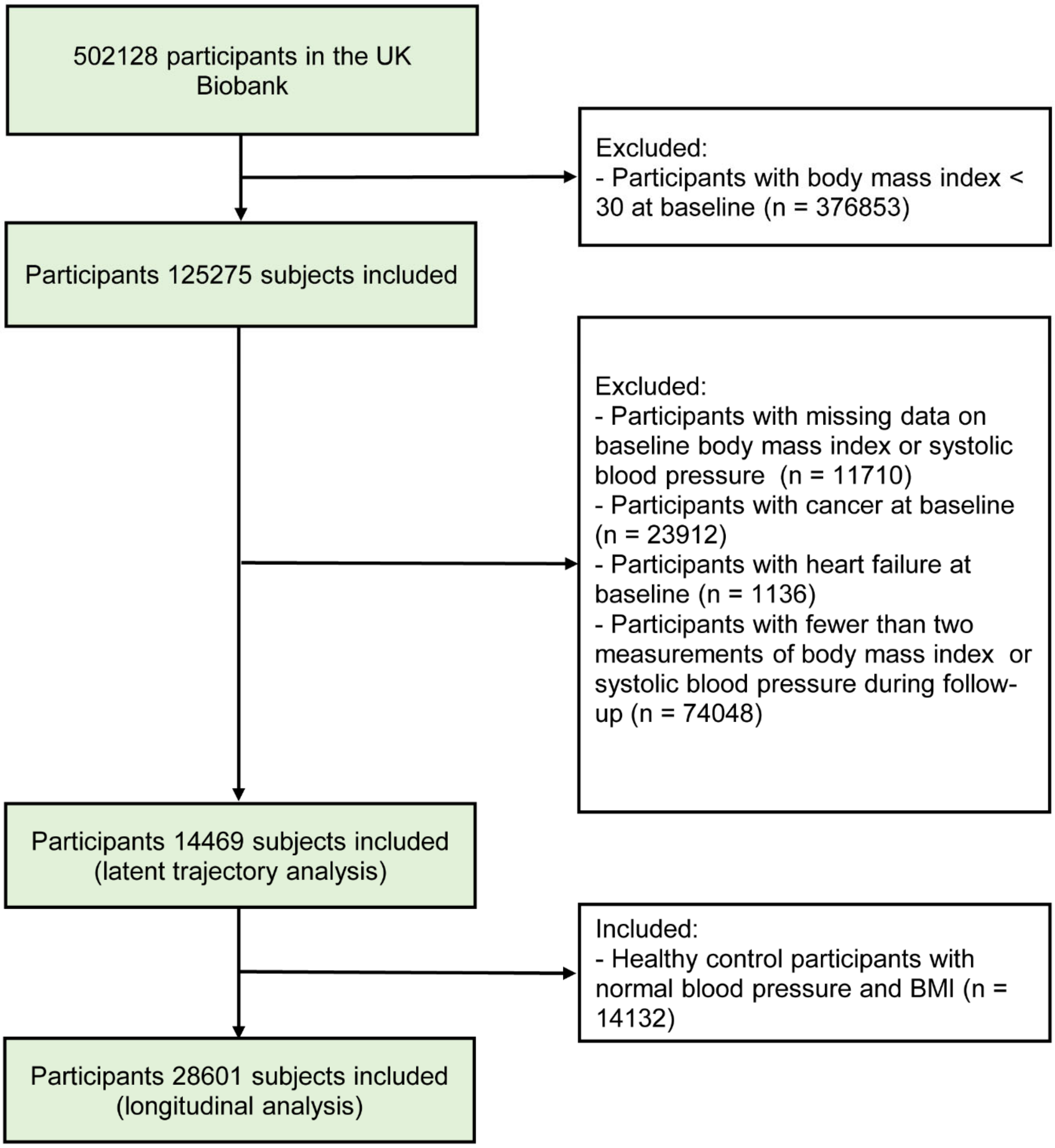

Figure S1. Flow chat of study participants

Section S2: Materials and methods

Table S1. The field IDs of all variables in UKB cohort.

Field ID	Variable

21003	Age
31	Sex
21001	Body mass index
4079	Diastolic blood pressure
4080	Systolic blood pressure
30750	Glycated haemoglobin
30690	Cholesterol
30760	HDL cholesterol
30780	LDL direct
30870	Triglycerides
30700	Creatinine
30720	Cystatin C
30710	C-reactive protein
30880	Urate
21000	Ethnic background
20117	Alcohol drinker status
20116	Smoking status
6138	Education
41270	Diagnoses - ICD10
41280	Date of first in-patient diagnosis - ICD10
41271	Diagnoses - ICD9
41281	Date of first in-patient diagnosis - ICD9
191	Date lost to follow-up
40000	Date of death
131354	Date I50 first reported (heart failure)
22420	LV ejection fraction
24103	LV ejection fraction
31060	Left ventricular ejection fraction
22421	LV end diastolic volume
24100	LV end diastolic volume
31061	Left ventricular end diastolic volume
22423	LV stroke volume
24102	LV stroke volume
31064	Left ventricular stroke volume
22424	Cardiac output
24104	LV cardiac output
24181	LV longitudinal strain global
53	Date of attending assessment centre
20001	Cancer code, self-reported

Section S3: Latent trajectory analysis

Table S2. Multivariate trajectory Group-Based Multi-Trajectory Modeling optimal model.

Trajectory group	AvePP	OCC	Prop	BIC	AIC
Group 1	1	1	1	584810	584731.9
Group 2	0.945672	27.78476	0.377497	563864.4	563699.5
Group 3	0.928109	23.21575	0.36084	554349	554097.3
Group 4	0.923905	37.36862	0.248531	542741.5	542403

AvePP, average posterior probability; OCC, odds of correct classification; Prop, proportion; BIC, bayesian information criterion; AIC, akaike information criterion.

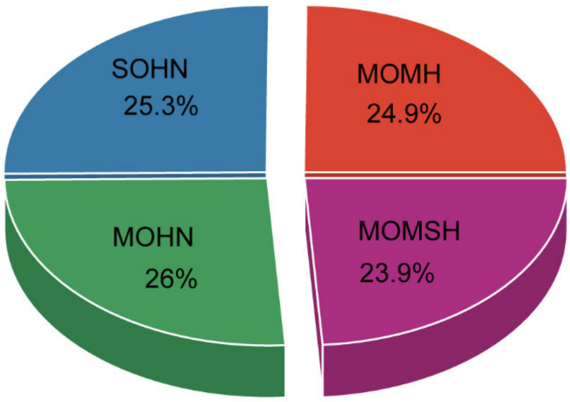

Figure S2. The pie chart of 4 trajectory subgroups. MOMH, mild obesity with mild hypertension trajectory; SOHN, severe obesity with high-normal blood pressure progression trajectory; MOHN, moderate obesity with high-normal blood pressure progression trajectory; MOMSH, mild obesity with moderate-to-severe hypertension improvement trajectory.

Section S4: Results

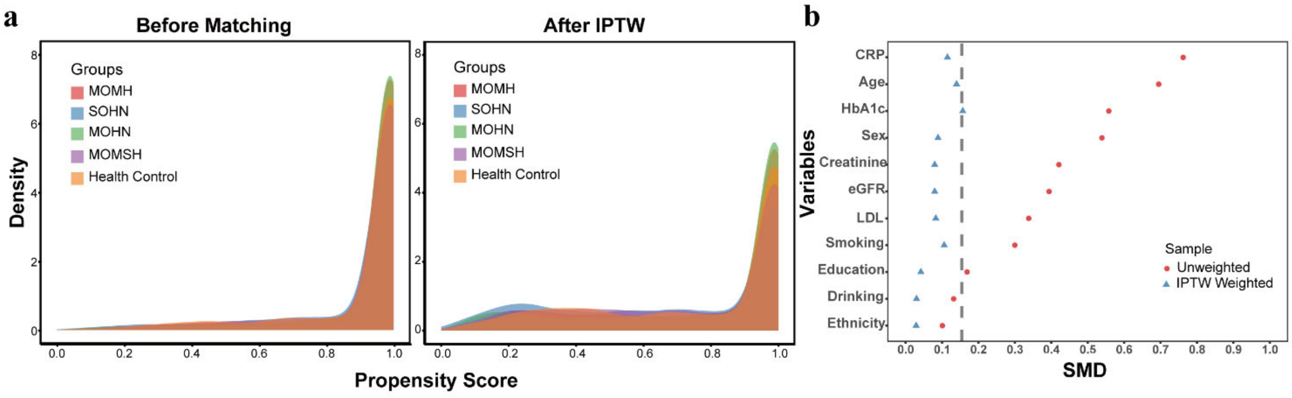

Figure S3. Evaluating covariate equilibrium pre- and post-IPTW adjustments. (a) Plot of standardized mean differences (SMD); (b) Density plot of propensity score distribution. IPTW, inverse probability of treatment weighting; MOMH, mild obesity with mild hypertension

trajectory; SOHN, severe obesity with high-normal blood pressure progression trajectory; MOHN, moderate obesity with high-normal blood pressure progression trajectory; MOMSH, mild obesity with moderate-to-severe hypertension improvement trajectory.

Table S3 Baseline characteristics of participants before IPTW matching.

Characteristics	MOMH	SOHN	MOHN	MOMSH	HC	*P*
Age, years	55.12 ± 7.28	52.79 ± 7.32	53.40 ± 7.27	57.52 ± 6.82	52.60 ± 7.32	<0.001
Sex, n (%)						<0.001
Female	1471 (40.9)	2332 (63.7)	1968 (52.4)	1344 (38.9)	9167 (64.9)	
Male	2125 (59.1)	1328 (36.3)	1787 (47.6)	2114 (61.1)	4965 (35.1)	
Ethnicity, n (%)						<0.001
White	3446 (95.8)	3491 (95.4)	3589 (95.6)	3364 (97.3)	13513 (95.6)	
Non-White	150 (4.2)	169 (4.6)	166 (4.4)	94 (2.7)	619 (4.4)	
Drinking status, n (%)						<0.001
Never	85 (2.4)	149 (4.1)	123 (3.3)	98 (2.8)	386 (2.7)	
Previous	90 (2.5)	148 (4.0)	99 (2.6)	75 (2.2)	317 (2.2)	
Current	3419 (95.1)	3359 (91.9)	3532 (94.1)	3282 (95.0)	13421 (95.0)	
Smoking status, n (%)						<0.001
Never	1970 (55.0)	2060 (56.5)	2106 (56.2)	1846 (53.6)	9274 (65.7)	
Previous	1384 (38.6)	1316 (36.1)	1364 (36.4)	1428 (41.5)	3859 (27.4)	
Current	231 (6.4)	269 (7.4)	279 (7.4)	170 (4.9)	974 (6.9)	
Education, n (%)						<0.001
College	415 (11.5)	386 (10.5)	421 (11.2)	425 (12.3)	2300 (16.3)	
Other levels	3164 (88.0)	3262 (89.1)	3319 (88.4)	3017 (87.2)	11793 (83.4)	
Unknown	17 (0.5)	12 (0.3)	15 (0.4)	16 (0.5)	39 (0.3)	
HbA1c, mmol/mol	36.48 ± 6.44	37.67 ± 8.15	36.70 ± 7.28	36.93 ± 6.55	33.95 ± 3.92	<0.001
LDL, mmol/L	3.66 ± 0.86	3.52 ± 0.84	3.61 ± 0.86	3.72 ± 0.90	3.43 ± 0.77	<0.001
eGFR, ml/(min×1.73m2)	94.84 ± 12.35	95.65 ± 13.49	95.77 ± 12.66	93.64 ± 12.15	98.41 ± 11.40	<0.001
CRP, mg/L	3.14 ± 4.11	4.63 ± 4.82	3.29 ± 4.19	3.02 ± 3.77	1.37 ± 3.22	<0.001

Creatinine, umol/L	75.07 ± 14.26	71.48 ± 14.57	73.20 ± 14.31	75.32 ± 14.42	68.95 ± 15.06	<0.001

Data are presented as mean ± standard deviation, or *n* (%). HbA1c, hemoglobin A1c; LDL, low-density lipoprotein; CRP, C-reactive protein; eGFR, estimated glomerular filtration rate; MOMH, mild obesity with mild hypertension trajectory; SOHN, severe obesity with high-normal blood pressure progression trajectory; MOHN, moderate obesity with high-normal blood pressure progression trajectory; MOMSH, mild obesity with moderate-to-severe hypertension improvement trajectory. * *P* <0.05.

Table S4 Comparison of covariates between groups after IPTW adjustment.

**Characteristics**	**MOMH**	**SOHN**	**MOHN**	**MOMSH**	**HC**	***P***
Age, years	53.92 ± 7.39	53.78 ± 7.39	53.74 ± 7.43	54.60 ± 7.23	53.57 ± 7.49	<0.001
Sex, n (%)						0.001
Female	1642.9 (53.1)	1705.8 (55.7)	1855.1 (55.8)	1502.1 (52.5)	7498.0 (56.9)	
Male	1451.1 (46.9)	1357.9 (44.3)	1469.7 (44.2)	1360.7 (47.5)	5682.2 (43.1)	
Ethnicity, n (%)						0.934
White	2949.9 (95.3)	2931.1 (95.7)	3189.6 (95.9)	2735.9 (95.6)	12596.1 (95.6)	
Non-White	144.0 (4.7)	132.5 (4.3)	135.2 (4.1)	126.8 (4.4)	584.1 (4.4)	
Drinking status, n (%)						0.983
Never	83.2 (2.7)	92.7 (3.0)	96.4 (2.9)	85.1 (3.0)	381.1 (2.9)	
Previous	81.6 (2.6)	87.8 (2.9)	81.2 (2.4)	68.3 (2.4)	330.3 (2.5)	
Current	2927.2 (94.7)	2877.8 (94.1)	3146.6 (94.7)	2707.2 (94.6)	12463.0 (94.6)	
Smoking status, n (%)						0.006
Never	1858.7 (60.2)	1744.2 (57.3)	1973.5 (59.4)	1766.1 (61.8)	8052.5 (61.2)	
Previous	1022.3 (33.1)	1066.0 (35.0)	1124.1 (33.8)	926.5 (32.4)	4143.3 (31.5)	
Current	205.0 (6.6)	236.0 (7.7)	223.2 (6.7)	162.9 (5.7)	962.5 (7.3)	
Education, n (%)						0.677
College	405.7 (13.1)	410.8 (13.4)	452.1 (13.6)	394.7 (13.8)	1910.0 (14.5)	
Other levels	2677.5 (86.5)	2643.4 (86.3)	2855.5 (85.9)	2458.1 (85.9)	11230.4 (85.2)	
Unknown	10.7 (0.3)	9.4 (0.3)	17.1 (0.5)	9.9 (0.3)	39.9 (0.3)	
HbA1c, mmol/mol	35.54 ± 5.57	35.76 ± 6.08	35.52 ± 5.75	35.65 ± 5.81	34.86 ± 4.83	<0.001

LDL, mmol/L	3.58 ± 0.81	3.53 ± 0.82	3.55 ± 0.81	3.59 ± 0.82	3.52 ± 0.81	0.002
eGFR, ml/(min×1.73m2)	96.37 ± 11.88	96.39 ± 12.03	96.16 ± 11.89	96.02 ± 12.06	97.00 ± 11.88	0.002
CRP, mg/L	2.56 ± 3.68	2.70 ± 3.78	2.53 ± 3.76	2.59 ± 3.62	2.23 ± 3.68	<0.001
Creatinine, umol/L	72.18 ± 13.42	71.73 ± 13.77	72.08 ± 13.25	72.33 ± 13.47	71.14 ± 15.18	<0.001

Data are presented as mean ± standard deviation, or *n* (%). HbA1c, hemoglobin A1c; LDL, low-density lipoprotein; CRP, C-reactive protein; eGFR, estimated glomerular filtration rate; MOMH, mild obesity with mild hypertension trajectory; SOHN, severe obesity with high-normal blood pressure progression trajectory; MOHN, moderate obesity with high-normal blood pressure progression trajectory; MOMSH, mild obesity with moderate-to-severe hypertension improvement trajectory. * *P* <0.05.

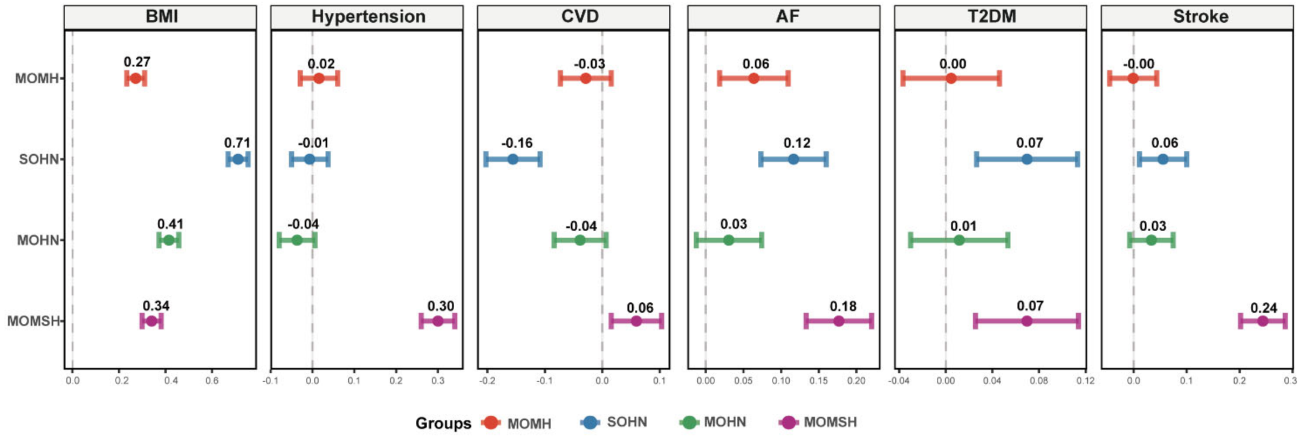

Figure S4. Polygenic risk score for different groups. Polygenic risk score are presented as mean ± standard. HC, health control; MOMH, mild obesity with mild hypertension trajectory; SOHN, severe obesity with high-normal blood pressure progression trajectory; MOHN, moderate obesity with high-normal blood pressure progression trajectory; MOMSH, mild obesity with moderate-to-severe hypertension improvement trajectory; BMI, body mass index; T2DM, type 2 diabetes mellitus; CAD, coronary artery disease; AF, atrial fibrillation; CAD, coronary artery disease.

Table S5 Sample size included in proteomic subgroup analysis.

Subgroup	N
HC	1575
MOMH	404
SOHN	412
MOHN	427
MOMSH	383

HC, health control; MOMH, mild obesity with mild hypertension trajectory; SOHN, severe obesity with high-normal blood pressure progression trajectory; MOHN, moderate obesity with high-normal blood pressure progression trajectory; MOMSH, mild obesity with moderate-to-severe hypertension improvement trajectory. Plasma proteomics analyses were performed using clusterProfiler (v4.10.1) and limma (v3.60.0).
